# Identification of a transcription factor, PunR, that regulates the purine and purine nucleoside transporter *punC* in *E. coli*

**DOI:** 10.1038/s42003-021-02516-0

**Published:** 2021-08-19

**Authors:** Irina A. Rodionova, Ye Gao, Anand Sastry, Ying Hefner, Hyun Gyu Lim, Dmitry A. Rodionov, Milton H. Saier, Bernhard O. Palsson

**Affiliations:** 1 Department of Bioengineering, University of California at San Diego, La Jolla, CA USA; 2 Department of Molecular Biology, Division of Biological Sciences, University of California at San Diego, La Jolla, CA USA; 3 Sanford-Burnham-Prebys Medical Discovery Institute, La Jolla, CA USA; 4 A.A. Kharkevich Institute for Information Transmission Problems, Russian Academy of Sciences, Moscow, Russia; 5 Novo Nordisk Foundation Center for Biosustainability, Technical University of Denmark, Lyngby, Denmark

**Keywords:** Bacterial genetics, Cell growth, Bacterial systems biology, Data mining

## Abstract

Many genes in bacterial genomes are of unknown function, often referred to as y-genes. Recently, the analytic methods have divided bacterial transcriptomes into independently modulated sets of genes (iModulons). Functionally annotated iModulons that contain y-genes lead to testable hypotheses to elucidate y-gene function. The inversely correlated expression of a putative transporter gene, *ydhC*, relative to purine biosynthetic genes, has led to the hypothesis that it encodes a purine-related transporter and revealed a LysR-family regulator, YdhB, with a predicted 23-bp palindromic binding motif. RNA-Seq analysis of a *ydhB* knockout mutant confirmed the YdhB-dependent activation of *ydhC* in the presence of adenosine. The deletion of either the *ydhC* or the *ydhB* gene led to a substantially decreased growth rate for *E. coli* in minimal medium with adenosine, inosine, or guanosine as the nitrogen source. Taken together, we provide clear evidence that YdhB activates the expression of the *ydhC* gene that encodes a purine transporter in *E. coli*. We propose that the genes *ydhB* and *ydhC* be re-named as *punR* and *punC*, respectively.

## Abstract (web summary)

Rodionova et al. find that the putative transporter gene, ydhC and its regulator ydhB are involved in purine transportation and that the expression of the ydhC gene is activated by the YdhB in *E. coli*. The authors suggest renaming the regulator PunR and the transporter PunC, respectively.

## Introduction

At least one-third of the genes in the growing number of available sequenced microbial genomes are still of unknown function. Furthermore, the functional annotations of a large portion of the remaining genes are based exclusively on sequence similarity to a relatively small pool of experimentally studied proteins. Even the best-studied model bacterium, *Escherichia coli* K-12 MG1655, is not an exception, despite the long-term effort to curate available knowledge-bases. EcoCyc is the premier example of a curated knowledge-base that summarizes functional evidence for over 4500 genes, including 1567 enzymes, 282 transporters and 204 transcription factors (TFs)^1^. Previous efforts to construct a global functional atlas of *E. coli* proteins revealed that 1431 of 4225 (35%) protein-coding genes were still not functionally annotated as of 2019^2,3^ based on three criteria: (i) the gene name starts with ‘y’ (hence, it is a y-gene); (ii) it does not have a linked pathway in EcoCyc; and (iii) it has a ‘predicted’, ‘hypothetical’ or ‘conserved’ protein functional description in GenProtEC^4^. The COMBREX knowledge base further reveals that experimental evidence is lacking for 44% of *E. coli* proteins^5^.

The bioinformatics prediction and experimental confirmation of microbial gene functions is a challenging problem. Bioinformatics analyses for the prediction of gene regulation, gene clustering and metabolic pathway reconstruction lead to reliable hypotheses, followed by experimental verification^6–8^. Such discovery efforts for specific classes of gene functions, such as TFs, are bearing fruit^9,10^, leading to a complete list of 278 TFs in *E. coli*.

The transcriptional regulation of purine/pyrimidine biosynthesis and the salvage regulon are coordinated by the transcriptional regulator PurR in Enterobacteria. We previously identified groups of genes using RNA-Seq data analysis, computed by independent component analyses applied to a large transcriptomic dataset, which were independently but coordinatively regulated (modulated) under different conditions compared to all other genes. Such coordinatively regulated groups of genes are called iModulons^11^. iModulons differ from regulons in that they are computed as independent source signals in a set of transcriptomic datasets (a dynamic measure). Regulons, on the other hand, are defined by TF binding sites in the promoters of the regulated genes (a static measure). iModulons show considerable overlap with previously characterized regulons^11^. In some cases, iModulons split such regulons into two dynamically regulated sets of genes, thus creating two iModulons corresponding to a single regulon.

**Fig. 1:**
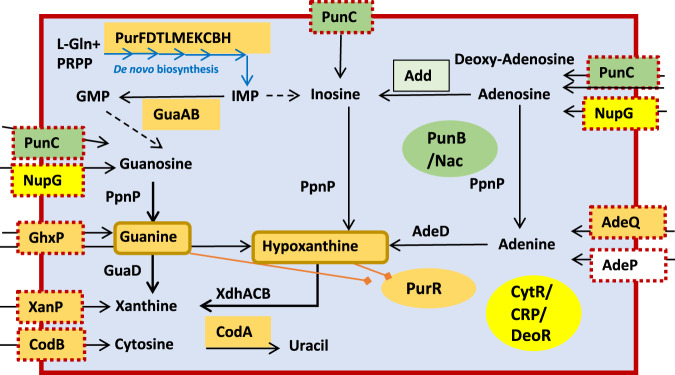
Metabolic pathways and transcriptional regulons for purine biosynthesis and purine/pyrimidine uptake and salvage genes in *E. coli*. The de novo purine biosynthesis pathway is illustrated with blue arrows. Other enzymes involved in purine and pyrimidine metabolism are shown with black arrows. Nucleoside uptake transporters are shown having dashed boxes. PurR is a transcriptional repressor of most of the purine biosynthesis and salvage genes in response to hypoxanthine and guanine. The PurR-regulated genes are highlighted in orange. The purine uptake transporter NupG is under transcriptional control of CytR, DeoR and CRP (shown in yellow). The purine transporter PunC (YdhC), described in this study, is controlled by the LysR-family activator PunR (YdhB) and Nac, as highlighted in green. Abbreviations: IMP, inosine monophosphate; AMP, adenosine monophosphate; GMP, guanosine monophosphate.

One example highlighted in this study is the PurR-1 iModulon, which contains genes related to purine biosynthesis from PRPP and utilization, as a part of the PurR regulon (Fig. 1)^11^. Genes encoding the purine biosynthetic pathway and the transporters (*ghxP*, *xanP*) were found to be repressed in the presence of exogenous adenine, whereas two genes were upregulated in the presence of adenine: *add*, encoding adenosine deaminase and involved in purine catabolism/salvage, and *ydhC*, encoding a putative transporter of unknown function, belonging to the Drug:H^+^ Antiporter-1 (DHA1) family (TCDB subfamily ID 2.A.1.2). The expression of these genes is inversely correlated with purine biosynthesis gene expression^11^. In particular, the *add* gene is directly activated by the ribose-responsive transcriptional regulator, RbsR, while the transcription of *purHD* is repressed by RbsR^12^, suggesting the dependency of purine biosynthesis on the availability of intracellular ribose.

The reconstruction of purine biosynthesis from 5-phospho-D-ribose-diphosphate and regulation is important for the gene function hypothesis. Independent component analysis (ICA) revealed the transcriptional regulation of the Pur-1 iModulon, consisting of the *xanP, purB, purC, purD, purE, cvpA-purF, purH, purK, purL, purM, purN, purT* and *ghxP* genes and those encoding the corresponding transporters. As well as the inverse regulation of the transcription of the *ydhC* and *add* genes in response to the presence of adenine in the growth medium had been detected by ICA^11^. Thus, purine biosynthesis pathway genes and those encoding the guanine/hypoxanthine and xanthine transporters (*ghxP*, *xanP*) are repressed by PurR in response to the availability of cellular hypoxanthine (Fig. 1), however, the regulatory mechanism for *ydhC* was not reported. YdhC had previously been detected in the *E. coli* BW25113 strain as a possible arabinose efflux transporter in rich medium using an arabinose cytoplasmic concentration reporter strain^13^. This suggests a possible relationship between the pentose cytoplasmic concentration and nutrient uptake from a rich medium; it is highly upregulated, specifically by adenine (iModulonDB).

The reconstruction of purine salvage and utilization under different conditions shows that adenosine/adenine and guanosine/guanine transporters are important as mediators of the uptake of purines when present in the medium as a supplement or substitute for a more traditional carbon or nitrogen source. Guanosine, inosine, cytidine and thymidine (but not uridine, adenosine and xanthosine) are transported by NupG^14^, and the pyrimidine nucleoside:H^+^ symporter, NupC, has been characterized^14,15^. NupG and NupC can recognize the nucleoside ribose moiety^14^ and are regulated by the global carbon catabolite repressor protein, CRP, as well as pyrimidine sensor/regulators, CytR and DeoR^16^. The *nupC* and *nupG* transporters transcriptionally activated by the presence of cytidine, the absence of glucose, but no difference for *nupG* and *nupC* expression was observed in the presence of adenine as a supplement to M9 medium (iModulonDB). NupC and NupG were shown recently to be important ADP-glucose uptake transporters, and they are essential for the incorporation of extracellular ADP-glucose into glycogen during biosynthesis^16^. The coordinate regulation of different purine/pyrimidine transporters is important for the utilization of nucleosides as carbon and nitrogen sources or for nucleoside salvage under various starvation conditions^17^.

Adenosine as a nitrogen source is utilized via adenosine deaminase (Add), which converts it to inosine (Fig. 1). PpnP is a broad specificity pyrimidine/purine nucleoside phosphorylase that produces hypoxanthine (a purine base) and D-ribose-1-phoshate from phosphate and inosine (the corresponding nucleoside), respectively^18^. However, it also acts on uridine, adenosine, guanosine, cytidine, thymidine and xanthosine as substrates. Adenosine utilization as a nitrogen source by the YdhC transporter, re-named PunC, and Add is proposed.

We hypothesize that PunC is an adenosine/adenine transporter, because the presence of a purine source in the medium inhibits biosynthesis (via PurR-dependent repression), while upregulating PunC, which had been shown to be highly upregulated during adenine supplementation in minimal medium^19,20^. Although NupG is a known purine transporter, regulation by CRP and CytR suggests that NupG is essential for uptake when a purine is to be used only as a carbon source. NupG requires hydroxyl groups in the ribose moiety for substrate binding and is not utilized for adenine uptake.

The *punC* (b1660) regulation by nitrogen assimilation protein (Nac) was observed by RNA-Seq according to published data^19^ (iModulonDB). Nac activates the pathways for the utilization of histidine, proline, urea and alanine in *Klebsiella pneumoniae*, a related Enterobacteria^20^. The physiological function of PunC likely allows supplementation of *E. coli* growth with a purine nucleobase as a nitrogen source or during purine salvage.

Here, we identify a LysR-family regulator, YdhB, and propose that YdhB directly controls the transcription of *punC* in response to adenine. We also provide evidence that PunC is a purine and purine nucleoside transporter (Fig. 1) and that it is important for resistance to sulfonamides. Machine learning-based analysis of RNA-Seq data was a useful tool for studying the co-expression of genes that identified the *ydhBC* genes as targets for investigation. The work described here expands our understanding of how purine uptake for salvage and degradation versus biosynthesis is reciprocally regulated in *E. coli*.

The PurR regulon is essential for purine biosynthesis when salvage is limited. PurR repression is activated by the presence of hypoxanthine or guanine, signalling that the purine cytoplasmic concentration is sufficient for growth^17,21,22^. The inverse correlation for the modulation of mRNA levels for PurR-regulated purine biosynthetic genes and those of the *ydhC* and *add* genes^23^ has been demonstrated during growth in minimal medium with glucose and adenine as supplements, suggesting a role in purine salvage^11^. However, no changes in mRNA level were found for *nupC* and *nupG* (iModulonDB).

## Results

### Systems analysis and the prediction of PunC function

The poor fitness phenotype for a *punC* mutant, encoding a homologous transporter in *Pseudomonas simiae* WCS417 (66% identity with the *E. coli* protein), has been demonstrated during growth in minimal medium, specifically with adenine as the nitrogen source (fit.genomics.lbl.gov). We suggested that the homologous *E. coli* transporter has adenine/adenosine specificity. The proposed regulation of the PurR-1 iModulon and reconstruction of purine biosynthesis and purine uptake is shown in Fig. 1. The reconstruction of purine biosynthesis and regulation is important for the gene function hypothesis. Extracellular adenosine/adenine potentially gives rise to hypoxanthine due to uptake followed by the action of adenosine deaminase, and further, by the PpnP-catalysed reaction (Fig. 1). However, an adenosine uptake transporter for nitrogen source utilization was not found.

### Prediction of PunR-binding sites

**Fig. 2:**
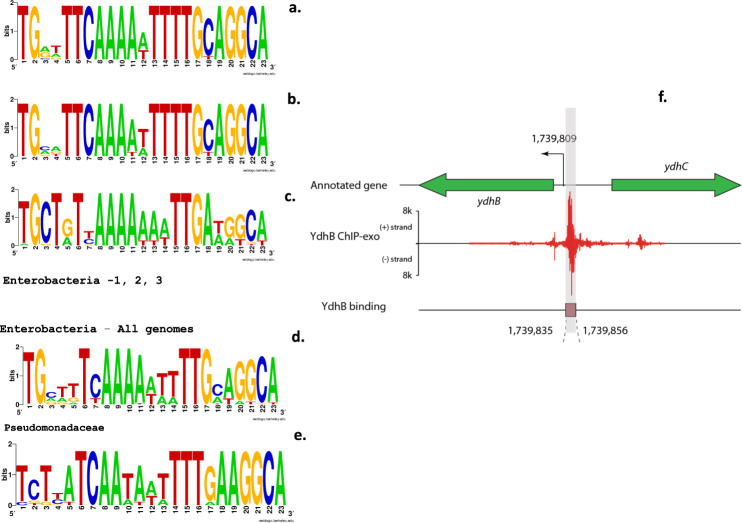
The predicted PunR-binding site motifs in various groups of Enterobacteria and *Pseudomonas* spp. **a**–**e** The predicted PunR-binding site motifs in various groups of Enterobacteria and *Pseudomonas* spp. as identified by a phylogenetic footprinting approach. **f** Identification of the PunR binding region using ChIP-exo. Sequence logos were generated based on the multiple alignments of PunR-binding sites from respective groups of Enterobacteria or *Pseudomonas* genomes (see Supplementary Fig. 2) using the WebLogo tool. The representative groups for Enterobacteria-1, 2, 3 and Pseudomonadaceae are described in Supplementary Fig. 2.

The previously uncharacterized transcriptional regulator YdhB (re-named PunR here) belongs to the LysR family of bacterial TFs. The *punR* gene is located in a conserved gene cluster with the divergently transcribed *punC* gene, and this arrangement is conserved in other Proteobacteria (Supplementary Fig. 1). The *punR* promoter is predicted to be Sigma 24-dependent, and the *punC* promoter is Sigma 24- and Sigma 70-dependent (RegulonDB). RpoD sigma factor binding upstream of *punC* has been experimentally detected^24^. To identify and characterize DNA-binding sites of PunR in the *E. coli* genome, we utilized combined bioinformatics and experimental approaches. We applied a comparative genomic approach of phylogenetic footprinting^25^ to predict the putative PunR-binding site in the common intergenic region between the *punR* and *punC* genes (Supplementary Fig. 2). Alignment of the upstream regions for the *punC* genes from three groups of Enterobacteria revealed conserved motifs with a common 23 bp palindromic consensus for binding of the predicted PunR regulator (Fig. 2). A similar palindromic motif was also identified in the *punC*/*punR* intergenic region in *Pseudomonas* spp. (Fig. 2), confirming strong conservation of the predicted PunR-binding site across gamma-Proteobacteria. A common motif of these orthologous operators is an imperfect palindrome with consensus TsttwTCAAwAwwwTTGaaGGCA, where ‘s’ is either G or C and ‘w’ is either A or T.

### Experimental validation of the PunR-binding site

We conducted ChIP-exo studies in M9 glucose medium to validate the predicted PunR operator and searched for additional candidate PunR-binding sites in the *E. coli* genome (Fig. 2 and Supplementary Table 1). The chromosomal *punR* gene was genetically modified to encode a Myc-tagged PunR. The *E. coli* strain was grown with glucose as the carbon source in M9 minimal media, and the ChIP-exo experiment was designed to pull down PunR, which occupied an ~200 bp region. The PunR-protected region from *E. coli* contained the predicted PunR-binding site upstream of *punC* (Fig. 2).

**Fig. 3:**
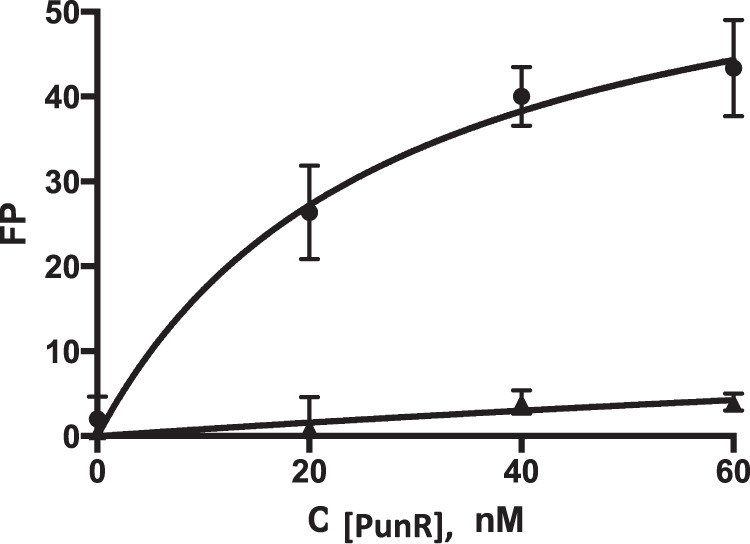
DNA binding assays with PunR. Binding of purified PunR protein to the predicted DNA sequence upstream of *punC* was assessed by the fluorescent polarization assay in the presence of 0.35 mM adenine.

To confirm PunR binding to its predicted operator site, we conducted fluorescence polarization (FP) and electrophoretic mobility shift assays (EMSA) with the purified PunR protein (40% purity). FP assays allow to detect binding between small fluorescently labelled DNA and large PunR protein molecules. Increasing concentrations of the PunR protein (0–60 nM) were incubated in the assay mixture with a fluorescently labelled 36 bp DNA fragment containing the candidate 23 bp DNA motif in the presence and the absence of adenine (Fig. 3). Using FP measurements, the target DNA fragment demonstrated the PunR concentration-dependent binding in the presence, but not in the absence, of 0.35 mM adenine. The PunR binding constant for binding to the DNA was calculated (*K*_*d*_ = 27 nM). The EMSA assay was further conducted with increasing concentrations of PunR (0–1500 nM) with the same fluorescently labelled DNA fragment in the presence of 1 and 10 mM of adenine. The disappearance of PunR-binding site DNA (fluorescently labelled) in the presence of adenine suggests binding of PunR. In contrast, no PunR binding to negative control-labelled DNA was detected (Supplementary Fig. 3).

### RNA-Seq analysis of the PunR regulon in adenosine or adenine supplemented media

RNA-Seq analysis of *E. coli* MG1655 (WT) in M9 with glucose as the carbon source (to exclude NupG overproduction), supplemented with 2.5 mM adenosine, showed more than a two-fold upregulation of the *punC* gene compared to the isogenic wild-type strain (Supplementary Data 1). The *punR* deletion strain of *E. coli* MG1655 showed a lower expression level of *punC* (Table 1), while the levels of other mRNAs were not changed in the presence of adenosine, suggesting that PunR is a specific activator for *punC* expression. The location of the PunR-binding site upstream of the predicted *punC* promoter agrees with the PunR-dependent activation of *punC* (Supplementary Fig. 2).

**Table 1:** RNA-Seq measurements for the differentially expressed gene, *punC* in *punR* deletion mutant strain compared to the *E. coli* MG1655 wild-type strain.

Gene name	Locus tag	Gene function (Uniprot)	Mean	log_2_ fold change	*P*-value
*punC*	b1660	Inner membrane transport protein	33.9	−2.55	1.05e-6

**Fig. 4:**
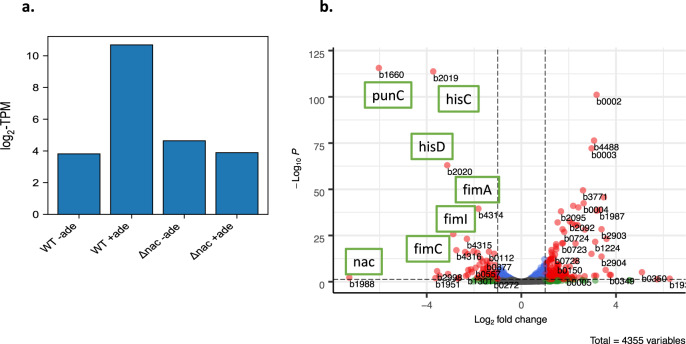
RNA-Seq analysis for *punC* mRNA expression. **a***E. coli* MG1655 and the *nac* mutant were grown in M9 medium in the absence or presence of 10 mM adenine. **b** mRNA differential expression for the *nac* mutant and wild-type in *E. coli* MG1655. Volcano plot comparing the log-fold expression change of genes against the adjusted p-value for the *nac* mutant and WT MG1655 grown at M9 supplemented with 10 mM adenine. Genes in red dots are differentially expressed. *punC* (b1660) is the gene with the strongest expression change in addition to *hisG* (b2019) and *hisD* (b2020) histidinol dehydrogenase (L-histidine biosynthesis), *fimA* (b4314), *fimI* (b4315) and *fimC* (b4316).

Upregulation of *punC* has been shown by RNA-Seq analysis according to published data^19^ for *E. coli* MG1655 (WT) in M9 minimal medium in the presence, but not in the absence, of adenine (iModulonDB). It was also revealed that *punC* regulation is Nac-dependent (in addition to PunR). The *punC* expression profile analyses revealed upregulation during growth in M9 medium supplemented with 10 mM adenine in the presence of Nac, but not in its absence (Fig. 4a), suggesting that both regulators are essential for *punC* transcriptional activation. The volcano plot for the differentially expressed genes compared to the *nac* mutant is shown in Fig. 4b. Supplementation of M9 medium with adenosine/adenine increased *punC* mRNA levels only in the presence, but not in the absence, of PunR/Nac (iModulonDB).

### Growth in M9 medium with adenosine or 2-deoxyadenosine as the nitrogen source and glucose or glycerol as the carbon source

**Fig. 5:**
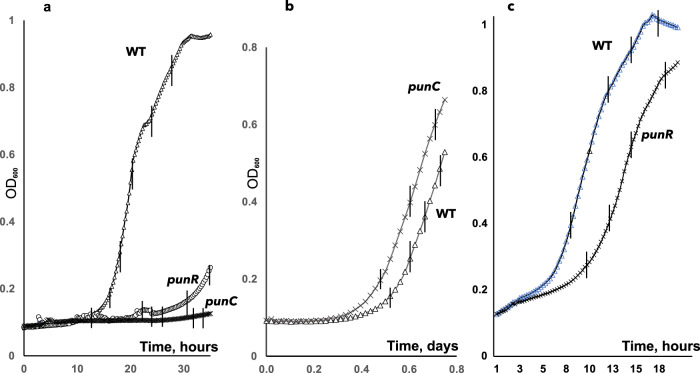
Growth phenotypes of the punR and punC knockout strains in M9 medium. Growth phenotypes of the *punR* and *punC* knockout strains in M9 medium with 0.2% glucose as the sole carbon source and **a** 2.5 mM adenosine as the sole nitrogen source, **b** NH_4_Cl as the nitrogen source. The growth of the *punR* (O) and *punC* (X) deletion strains compared to the wild-type *E. coli* BW25113 (Δ) strain. **c** Growth of *E. coli* MG1655 wild-type (Δ) and *punR* (O) deletion strain in the same medium as in (**a**).

Two *E. coli* strains, MG1655 and BW25113 (Keio collection of single-gene knockouts), have been tested with glucose as the carbon source, when adenosine was added to the M9 medium. The differences between these two strains have been revealed by iModulon analysis^11^. Under nitrogen-limiting conditions, growth of *E. coli* BW25113 was possible, but no growth was observed for the *punR* or *punC* mutants, even after 20 h (Fig. 5a). However, growth in M9 medium with NH_4_Cl for the *punC* mutant was detected (Fig. 5b). The *punR* mutant of *E. coli* MG1655 had a negative growth-phenotype under the same conditions (Fig. 5c). When the *nupG* mutant was tested with the same M9 medium (2.5 mM adenosine as the nitrogen source), no growth-phenotype was observed.

**Fig. 6:**
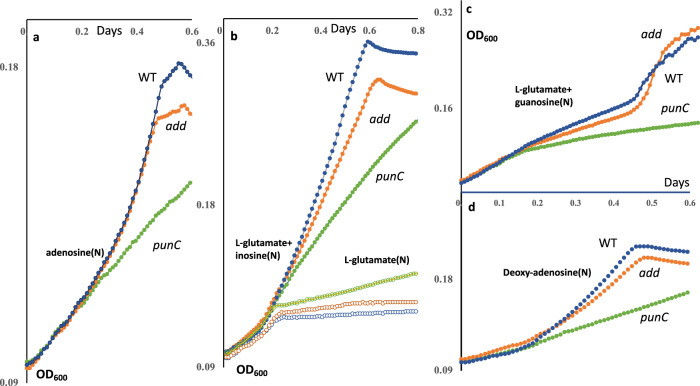
Growth phenotypes of the *add* and *punC* knockout strains in M9 medium with 0.4% glycerol as the carbon source and various nitrogen sources as follows. **a** 2.5 mM adenosine as the sole nitrogen source, **b** 5 mM L-glutamate and inosine, or 5 mM L-glutamate (empty circles with strains indicated with corresponding colour), **c** 5 mM L-glutamate and guanosine and **d** 5 mM 2-deoxyadenosine as the sole nitrogen source. The growth of the *punC* (green circles) and *add* (orange line, circles) deletion strains were compared to the wild-type *E. coli* BW25113 strain (dark blue line, circles).

When the *punC* and *add* mutants of *E. coli* BW25113 were examined with 0.4% glycerol as the carbon source and 2.5 mM adenosine as the nitrogen source, the growth effect was substantial (the growth phenotypes are shown in Fig. 6a). The growth phenotype with 5 mM 2-deoxyadenosine for the *punC* mutant was determined, while the *add* deletion had no effect on growth. These effects are shown in Fig. 6d. We suggest that high concentrations of adenosine/2-deoxyadenosine can support metabolism via the pentose phosphate pathway under nitrogen/carbon starvation, and that PunC is the major transporter for the nucleoside supporting growth under these conditions.

### Growth in M9 medium with guanosine, inosine or glutamate as the nitrogen source and glycerol as the carbon source

Supplementation of M9 minimal medium with glycerol as the sole carbon source and glutamate as the nitrogen source did not support *E. coli* growth (Fig. 6). The effect of either inosine or guanosine as the supplemental nitrogen sources was examined using the WT and isogenic *add* and *punC* deletion mutant strains, measuring growth when glycerol was the carbon source. The *punC* mutant showed a decrease in the growth rate compared to the WT strain under nitrogen starvation conditions when inosine or guanosine was present as an additional nitrogen source (Fig. 6b, c). This suggested that the PunC transporter can take up, in addition to adenosine and 2-deoxyadenosine, inosine and guanosine. Thus, the PunC uptake transporter exhibits broad specificity for purine nucleosides as revealed by these phenotypic analyses. The *punC* homologue deletion mutant in *Klebsiella michiganensis* showed a decrease in the growth fitness in defined medium when adenosine, inosine, 2-deoxyadenosine, 2-deoxyinosine and 2-deoxyadenosine-phosphate were carbon sources, but not for other 189 experimental conditions including different carbon or nitrogen sources (fit.genomics.lbl.gov).

### PunC specificity screening for carbon sources using Biolog plate 1 with NH_4_Cl as the nitrogen source

The growth/respiration observed for wild-type (WT) *E. coli* BW25113 and its isogenic *punC* mutant using different carbon sources, including adenosine or inosine, on Biolog plate PM1 (biolog.com), was measured. The strains were grown overnight in LB and washed twice with M9 medium without the addition of a carbon source. The cultures were diluted in M9 medium without a carbon source, and 0.1 ml of each was added to the 96-well plates. Growth/respiration was detected with Omnilog, but very little difference with adenosine, 2-deoxyadenosine, D-glucose, D-mannitol, D-fructose, D-xylose or other carbon sources was detected for the mutant and WT strains under microaerobic conditions (Supplementary Fig. 4). Growth/respiration of the *punC* deletion mutant in minimal medium with adenosine as the carbon source was likely supported by an alternative transporter.

### PunC transporter specificity screening for antibiotic sensitivities using Biolog plates 11C and 12

**Fig. 7:**
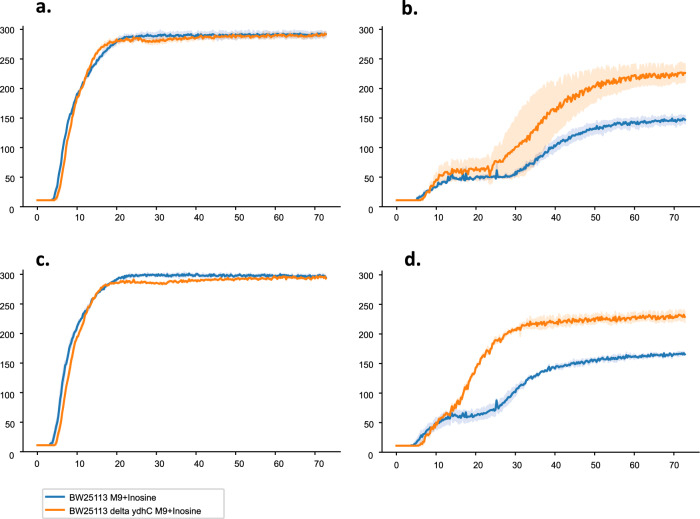
The phenotypes of the *E. coli* BW25113 and *punC* knockout strains in the Biolog plate PM12B supplemented with 1 mM inosine. Resistance to: **a**, **b** sulfathiazole_1X and 8X, and **c**, **d** sulfamethoxazole_1X and 8X (folate antagonists). The other antibiotics tested at four different concentrations are penicillin G, tetracycline, carbenicillin, oxacillin, penimepicyclin, polymyxin B, paromomycin, vancomycin, D,L-serine hydroxamate, sisomycin, sulfamethazine, novobiocin, 2,4-diamino-6,7-diisopropyl-pteridine, sulfadiazine, benzethonium chloride, tobramycin, 5-fluoroorotate, spectinomycin, L-aspartic-b-hydroxamate, spiramycin, rifampicin and dodecyltrimethyl ammonium bromide.

We screened for the possible PunC-mediated transport of various antibiotics using the *punC* mutant compared to the *E. coli* BW25113 (WT) strain. For these purposes, we used Biolog plates 11C and 12. Each of these plates contained four different concentrations of 24 different antibiotics. The *punC* mutant (kanamycin resistant) and WT strains showed the same resistance for all of the included antibiotics except paromomycin, 2,4-diamino-6,7-diisopropyl-pteridine, L-aspartic-β-hydroxamate and sulfonamides: sulfathiazole, sulfadiazine and sulfamethoxazole. The inhibition effect for kanamycin has not been detected for the wild-type strain. The other antibiotics tested at four different concentrations were penicillin G, tetracycline, carbenicillin, oxacillin, penimepicyclin, polymyxin B, paromomycin, vancomycin, D,L-serine hydroxamate, sisomycin, sulfamethazine, novobiocin, 2,4-diamino-6,7-diisopropyl-pteridine, sulfadiazine, benzethonium chloride, tobramycin, 5-fluoroorotate, spectinomycin, L-aspartic-β-hydroxamate, spiramycin, rifampicin, dodecyltrimethyl ammonium bromide, amikacin, chlortetracycline, lincomycin, amoxicillin, cloxacillin, lomefloxacin, bleomycin, colistin, minocycline, capreomycin, demeclocycline, nafcillin, cephazolin, enoxacin, nalidixic acid, chloramphenicol, erythromycin, neomycin, cephtriaxone, gentamicin, hydroxamate, cephalothin, kanamycin and ofloxacin. The *punC* mutant resistance effect was observed at different sulfonamide concentrations (Fig. 7). The increased resistance observed for the *punC* deletion mutant strain suggests that this transporter exhibits specificity for the uptake of sulfonamides. The sulfonamide group of antibiotics are structural analogues of para-aminobenzoic acid (PABA), an intermediate of folate biosynthesis. Mercaptoguanine derivatives inhibit the conversion of PABA and 6-hydroxy-methyldihydropterin-PP to dihydropteroate^26^. In contrast, the *punC* mutant was inhibited by 2,4-diamino-6,7-diisopropyl-pteridine (folate antagonist) and L-aspartic-β-hydroxamate substantially compare to the wild-type strain (Fig. 7). It is interesting that a distant *punC* paralogous gene in *E. coli* encodes Bcr—a bicyclomycin/sulfonamide resistance protein^27^. Bcr has been shown to be involved in the export of L-cysteine^28^. In contrast, PunC appears to be an adenine/2-deoxyadenosine/adenosine/guanosine/inosine uptake transporter with broad purine specificity that may allow uptake of sulfamethoxazole and sulfothiazole, but not sulfamethazine. The analysis of *E. coli* inhibition by sulfonamide structural analogues shows the PunC specificity for different sulfonamides modifications (Fig. 7).

## Conclusion

A new RNA-Seq data analytic approach was applied to study the PurR regulon and the PurR-1 iModulon (see iModulonDB.org). The inverse relationship in the PurR-1 i-Modulon, between the expression of purine biosynthetic genes and *punC*, led to the hypothesis that *punC* encodes a purine uptake transporter. We addressed this hypothesis and showed that: (1) expression of the putative transporter, PunC, is essential for adenosine, inosine, guanosine and 2-deoxyadenosine uptake/growth under nitrogen starvation conditions, but not for growth using adenosine as a carbon source in M9 medium; (2) the upregulation of the *punC* gene in the presence of adenine in M9 medium is dependent on the nitrogen assimilation regulatory protein, Nac. The analysis of RNA-Seq data for the *nac* mutant during growth in the medium supplemented with 10 mM adenine showed the activation of *punC* only in the presence of *nac*. *PunC* is additionally regulated by the LysR family HTH-type transcriptional regulator PunR, and the *punR* gene is conserved within the *punC* genome context. The *punC* transporter is upregulated under nitrogen starvation conditions, consistent with the sigma-24 (sigma-E)-dependent promoter catalogued in RegulonDB^29^; and (3) The PunC-dependent uptake of sulfathiazole and sulfamethoxazole was inferred from the fact that the *punC* mutant strain has substantially increased resistance under microaerobic conditions compared to wild-type *E. coli* BW25113.

The *E. coli punC* deletion mutant has a growth defect in a minimal medium with either 2-deoxyadenosine, adenosine, inosine or guanosine as the nitrogen source. A *punC* mutant was found to be more resistant to representative toxic nucleobase analogues, suggesting an uptake function. The predicted DNA-binding of PunR to the palindromic sequence between *punR* and *punC*, conserved in many Proteobacteria, established it as the PunR-binding site. PunR binding was demonstrated using ChIP-exo and a fluorescent polarization assay and confirmed by EMSA. We suggest that PunR functions as an activating transcriptional regulator. This hypothesis is supported by the RNA-Seq analysis of the *punR* mutant and the fact that PunR binding has been detected in the presence, but not in the absence, of adenine by the FP method. The *punC* gene is present in many Proteobacterial genomes, including all Enterobacteria. The same is true for the *punR* and *purR* genes, which are conserved in the same gene contexts (Supplementary Fig. 1). It is interesting that PunR had previously been shown to be essential for *Yersinia pseudotuberculosis* growth^30^, suggesting that purine uptake may be a property of many Proteobacteria. The *Y. pseudotuberculosis* NupG transporter ortholog is absent, but the NupC ortholog is present. Additionally, adenosine as the sole nitrogen source can support *E. coli* growth, and it may be involved in acid resistance because the deletion of *add* attenuates growth in the presence of adenosine under acidic conditions^31^.

Adenine, adenosine and deoxyadenosine can be converted to the guanine nucleotide via the salvage pathway, which supports growth in minimal medium^32^. The presence of inosine or guanosine in the *E. coli* growth minimal medium increased the growth rate when used as a poor nitrogen source as with glutamate and glycerol (sole carbon source), and PunC is essential for this growth. The fitness phenotype for a *punC* mutant, encoding a homologous transporter in *P. simiae* WCS417, has been demonstrated with adenine as the nitrogen source^33^, but it proved to be only mildly important with adenosine as the nitrogen source (fit.genomics.lbl.gov). The PunC broad specificity function in *K.* *michiganensis* M5al is supported by the strong fitness phenotype for the *punC* mutant homologue (75% identity with the *E. coli* homologue). This mutant was found to have a strong negative fitness during growth with any one of several purines and purine nucleosides as carbon sources: inosine, 2-deoxyinosine, 2-deoxyadenosine and 2-deoxyadenosine 5-phosphate, as well as a mild negative effect with adenosine as the carbon source. All of these observations substantiate the main conclusion of this paper that PunC is an adenosine/inosine transporter in *E. coli*, but the PunC homologous transporter in *K.* *michiganensis* likely has broader specificity for 2-deoxyinosine/2-deoxyadenosine and 2-deoxyadenosine-phosphate.

Taken together, the data analysis approach used here shows that PunC family representatives are regulated by PunR and Nac. The transcriptional analysis revealed that the putative transporter encoded by *punC* is upregulated by Nac and correlates with PurR regulation. It is interesting that Nac also upregulates L-histidine biosynthetic genes and pilus synthesis in M9 supplemented with adenine (Fig. 4).

The approach for the ‘big data analysis’ applied in this paper produced a useful hypothesis about the functions of a y-gene transporter and a LysR-family regulator. The screening of data represented in the iModulonDB, RegulonDB, EcoCyc, Fitness Browser and PubSEED databases/platforms will help to produce new hypotheses for y-gene functions.

## Methods

### Bacterial strains and growth conditions

The *punR* mutant of the *E. coli* MG1655 strain was constructed as described in^9^. The *punR*, *add* and *punC* mutant strains of *E. coli* BW25113, from the Keio single-gene knockout collection^34^, or the *punR* mutant of *E. coli* MG1655 and the isogenic wild-type strain were grown overnight in LB medium at 37 °C. The *punR, add* and *punC* gene deletions were confirmed by polymerase chain reaction (PCR) using gene-specific primers. The cells were refreshed for 3 h, washed with M9 salts medium and inoculated in the M9 medium with 0.4% glycerol or 0.2% glucose as the carbon source and lacking the usual a nitrogen source, but substituting it with 2.5 mM adenosine, 5 mM 2-deoxyadenosine, 5 mM L-glutamate or 5 mM inosine or guanosine. The *E. coli* BW25113 wild-type and *punC* mutant strains were grown overnight in LB medium, washed with M9 medium and inoculated as recommended on to the Omnolog plates 1, 11C or 12B, for the antibiotic resistance measurements at 37 °C. The experiments were repeated three times.

### Identification of putative PunR-binding sites by comparative genomics

The potential PunR-binding sites were identified by a phylogenetic footprinting approach using multiple sequence alignments (Supplementary Fig. 2). Orthologs of the *E. coli punC* and *punR* genes in other Proteobacteria, as well as multiple sequence alignments of orthologous upstream regions, were identified using the PubSEED comparative genomics platform^35^. PunR-binding site sequence logos were constructed using the WebLogo tool^36^.

### RNA sequencing

RNA sequencing data were generated after growth under aerobic exponential growth conditions in M9 medium supplemented with adenosine. The wild-type MG1655 strain was grown as a control for the isogenic *punR* mutant strain. Pre-cultures for the RNA sequencing experiments were started for growing the cells in LB medium. Cells were then washed twice with M9 medium and inoculated at an OD_600_ of 0.05. The cells were collected at an OD_600_ of 0.6 and were harvested using the Qiagen RNA-protect bacterial reagent according to the manufacturer’s specifications. Pelleted cells were stored at −80 °C, and after cell resuspension and partial lysis, they were ruptured with a bead beater; the total RNA was extracted using a Qiagen RNA purification kit. After total RNA extraction, the quality was assessed using an Aglient Bioanalyzer using an RNA 6000 kit after removal of ribosomal RNA. Paired-end-strand-specific RNA sequencing libraries were prepared as described^9^.

Raw-sequencing reads were collected from GEO and mapped to the reference genome (NC_000913.3) using bowtie (v1.1.2) with the following options ‘-X 1000 -n 2 -3 3’. Transcript abundance was quantified using *summarizeOverlaps* from the R *GenomicAlignments* package (v1.18.0) with the following options: ‘mode = ‘IntersectionStrict’, singleEnd = FALSE, ignore.strand = FALSE, preprocess.reads = invertStrand’. To ensure the quality of the compendium, genes shorter than 100 nucleotides and genes with under 10 fragments per million-mapped reads across all samples were removed before further analysis. Transcripts per million (TPM) were calculated by DESeq2 (v1.22.1). The final expression compendium was log-transformed log_2_(TPM + 1) before analysis, erred to as log-TPM. Biological replicates with *R*^2^ < 0.9 between log-TPM were removed to reduce technical noise.

### ChIP-exo experiments

The strains harbouring 8-myc were generated by a λ red-mediated site-specific recombination system, targeting the C-terminal region as described previously^37^. ChIP-exo experimentation was performed following the procedures previously described^10,38^. To identify PunR-binding sites for each strain, the DNA bound to PunR from formaldehyde cross-linked cells, collected after growth in M9 supplemented with adenosine, was isolated by chromatin immunoprecipitation (ChIP) with the antibodies that specifically recognize the myc tag (9E10, Santa Cruz Biotechnology), and Dynabeads Pan Mouse IgG magnetic beads (Invitrogen) were added, followed by stringent washings as described previously^9^. ChIP materials (chromatin-beads) were used to perform on-bead enzymatic reactions of the ChIP-exo method. Briefly, the sheared DNA of the chromatin-beads was repaired by the NEBNext End Repair Module (New England Biolabs), followed by the addition of a single dA overhang and ligation of the first adaptor (5ʹ-phosphorylated) using a dA-Tailing Module (New England Biolabs) and NEBNext Quick Ligation Module (New England Biolabs), respectively. Nick repair was performed by using the PreCR Repair Mix (New England Biolabs). Lambda exonuclease- and RecJf exonuclease-treated chromatin was eluted from the beads, and overnight incubation at 65 degrees reversed the protein–DNA cross-link. RNA- and protein-free DNA samples were used to perform primer extension and second adaptor ligation with the following modifications. The DNA samples, incubated for primer extension as described previously^9^, were treated with the dA-Tailing Module and NEBNext Quick Ligation Module (New England Biolabs) for second adaptor ligation. The DNA sample, purified using the GeneRead Size Selection Kit (Qiagen), was enriched by PCR using Phusion High-Fidelity DNA Polymerase (New England Biolabs). The amplified DNA samples were purified again with a GeneRead Size Selection Kit (Qiagen) and quantified using Qubit dsDNA HS Assay Kit (Life Technologies). The quality of the DNA sample was checked by running the Agilent High Sensitivity DNA Kit using an Agilent 2100 Bioanalyzer before sequencing using HiSeq 2500 (Illumina) following the manufacturer’s instructions. Each modified step was also performed following the manufacturer’s instructions. ChIP-exo experiments were performed in duplicate.

### Overproduction of PunR protein

The *punR* overexpressing strain of *E. coli* was inoculated from the ASKA collection^39^ onto LB agar plates containing chloramphenicol. Overnight cultures were then inoculated from single colonies. Each of the new cultures (50 ml) was started, and after the OD_600_ reached 0.8 at 37 °C, 0.8 mM IPTG was added. The cultures were incubated at 24 °C overnight with continuous shaking, and cells were collected by centrifugation. The PunR recombinant protein containing an N-terminal His_6_ tag was purified by Ni-chelation chromatography from the soluble fraction as described^40,41^. The insoluble fraction was solubilized in 7 M urea and purified on a Ni-NTA mini-column with At-buffer (50 mM Tris-HCl buffer, pH 8, 0.5 mM NaCl, 5 mM imidazole and 0.3% Brij) with 7 M urea. Refolding of PunR protein was conducted on the column in At-buffer.

### In vitro DNA binding assays

The interaction of the purified PunR protein with its specific DNA-binding site was assessed using two approaches: (i) fluorescent polarization and (ii) EMSA with fluorescently labelled 36 bp DNA fragment (5ʹ-TGGggactggtttcaaaaattttgcaggcagagGGG), containing the predicted PunR-binding site and flanked on each side by extra guanine residues (capital case). As a negative control, a 26 bp DNA fragment containing the GguR binding site from *Polaromonas* sp. JS666 was used (5ʹ-ACCCCcagtcatcagacaacctCCCC)^42^. For each DNA fragment, two complimentary single-stranded oligonucleotides were synthesized by IDT, at that 5ʹ-3ʹ fragments were labelled by 6-carboxyfluorescein at 5ʹ end (underlined T or A). The double-stranded DNA fragment were obtained by annealing the labelled oligonucleotides with unlabelled complementary oligonucleotides at a 1:10 ratio. The obtained fluorescently labelled DNA fragments (10 nM) were incubated for 1 h at 30 °C with the increasing concentrations of PunR protein in the assay mixture (0.1 ml) in 96-well black plates. The binding buffer contained 20 mM Tris-HCl (pH 7.5), 0.1 M NaCl, 0.5 mM EDTA, 10 mM MgSO_4_, 2 mM DTT, 5 μg/ml herring sperm DNA. The fluorescence-labelled DNA was detected with the Beckman Coulter DTX 880 Multimode Detector. The effect of adenine (0–0.6 mM) on PunR binding to DNA was tested by its addition to the incubation mixture. For EMSA, the same fluorescently labelled DNA fragments (100 nM) were incubated increasing concentrations of PunR (0–1.5 μM) in the PBS (pH 8) buffer containing 10 mM MgSO_4_, 2 mM DTT and 0.25 mM adenine for 1 h at 30 °C. After 1 h of incubation at 25 °C, the reaction mixtures were separated by electrophoresis on the 6% polyacrylamide native gel in the Tris-glycine running buffer with 0.25 mM adenine. The fluorescently labelled DNA was detected with the BioRad ChemiDoc imaging system.

### Reporting summary

Further information on research design is available in the Nature Research Reporting Summary linked to this article.

## Supplementary information

Supplementary information.

Description of Additional Supplementary Files.

Supplementary Data 1.

Supplementary Data 2.

Reporting summary.

## Data Availability

The whole dataset of ChIP-exo and RNA-Seq has been deposited to GEO with the accession numbers of GSE166465 and GSE173410, respectively.
