# Supplementary material for: Identification of a transcription factor, PunR, that regulates the purine and purine nucleoside transporter punC in E. coli
Source: Commun Biol. 2021 Aug 19;4:991. doi: 10.1038/s42003-021-02516-0 (PMC8376909; doi:10.1038/s42003-021-02516-0)
Supplement: Supplementary file 1 — Supplementary information. [file 42003_2021_2516_MOESM1_ESM.pdf]

Supplementary Table and Figures

Supplementary Table 1. The ChIP-exo detected the PunR occupied an ~200 bp region in the promoter/ intergenic region for the listed genes.

Supplementary data. RNA-Seq measured differentially expressed genes in the presence and absence of 2.5 mM adenosine compared for *E. coli* MG1655 wild type strain.

Supplementary Figure 1. The genome context analysis of the *ydhC-ydhB* genes. Orthologous genes are marked by arrows of the same color and labeled according to their functional annotations: 3-PunC (YdhC), 1-PunR (YdhB), 2-PurR (purine nucleotide synthesis repressor).

Supplementary Figure 2. Multiple alignments of intergenic regions of the *ydhC/ydhB* genes in various groups of Enterobacteria and *Pseudomonas* spp. Gene coding regions are shown in red capital letters. Location of predicted PunR binding site is highlighted in yellow. The predicted -35 and -10 promoter elements of *punC* are underlined.

Supplementary Figure 3. The EMSA binding assay for PunR. The fluorescently labelled DNA pre-incubated for 1 hour with PunR and separated on 6% polyacrylamide gel. The lines -1, 2, 3 represent 1.5, 0.8, 0 microM of PunR incubated with the negative control – GguR- regulator fluorescently labelled DNA sequence in the presence of 1mM adenine. The lines 4, 5, 6, 7 represent PunR-predicted labelled DNA sequence incubated with PunR -0, 0.4, 0.8 and 1.5 microM in the presence of 1 mM adenine and lines 8, 9, 10 are PunR pre-incubated with the predicted PunR DNA-binding site at the 1, 0.5, 0 microM in the presence of 10 mM adenine. The Tris-Glycine running buffer, pH 8 supplemented with 0.25 mM adenine.

Supplementary Figure 4. The **phenotypes of the *E. coli* BW25113 and *punC* knockout strains in** the Biolog plate PM1 using M9 medium (PM1- carbon sources). The Omnilog measured growth curves for *E. coli* BW25113 and *punC* knockout strains on 2-deoxy-adenosine, adenosine, D-glucose, D-sorbitol, D-glucuronate, D-xylose. The Biolog plate PM1 contains 95 different carbon sources: L-arabinose, N-acetyl-D-glucosamine, D-saccharic acid, succinate, D-galactose, L-aspartate, L-proline, D-alanine, D-trehalose, D-mannose, dulcitol, D-serine, D-sorbitol, glycerol, L-fucose, D-glucuronate, D-gluconate, D,L-glycerol-phosphate, D-xylose, L-lactate, formate, D-mannitol, L-glutamate, D-glucose-phosphate, D-galactonate-lactone, D,L-malate, D-ribose, Tween 20, L-rhamnose, D-fructose, acetate, D-glucose, maltose, melibiose, thymidine, L-asparagine, D-aspartate, D-glucosaminate, 1,2-propandiol, Tween 40, α-keto-glutarate, α-keto-butyrate, α-Methyl-galactoside, D-lactose, lactulose, sucrose, uridine, L-glutamine, m-Tartrate, D-glucose-1-phosphate, D-fructose-6-phosphate, Tween 80, α-hydroxy-glutarate-lactone, α-hydroxy-butyrate, β-methyl-D-glucoside, adonitol, maltotriose, 2-Deoxy-adenosine, adenosine, glycyl-L-aspartate, citrate, Myo-inositol, D-threonine, fumarate, bromo-succinate, propionate, mucic acid, glycolate, glyoxylate, D-cellobiose, inosine, glycyl-L-glutamate, tricarballylate, L-serine, L-threonine, L-alanine, L-alanyl-glycine, acetoacetate, N-acetyl-D-mannosamine, mono-methyl-succinate, methyl-pyruvate, D-malate, L-malate, glycyl-L-proline, p-hydroxy-phenylacetate, m-hydroxy-phenylacetate, tyramine, D-psicose, L-lyxose, glucuronamide, pyruvate, L-galactonate-γ-lactone, D-galacturonate, phenylethylamine, 2-aminoethanol.

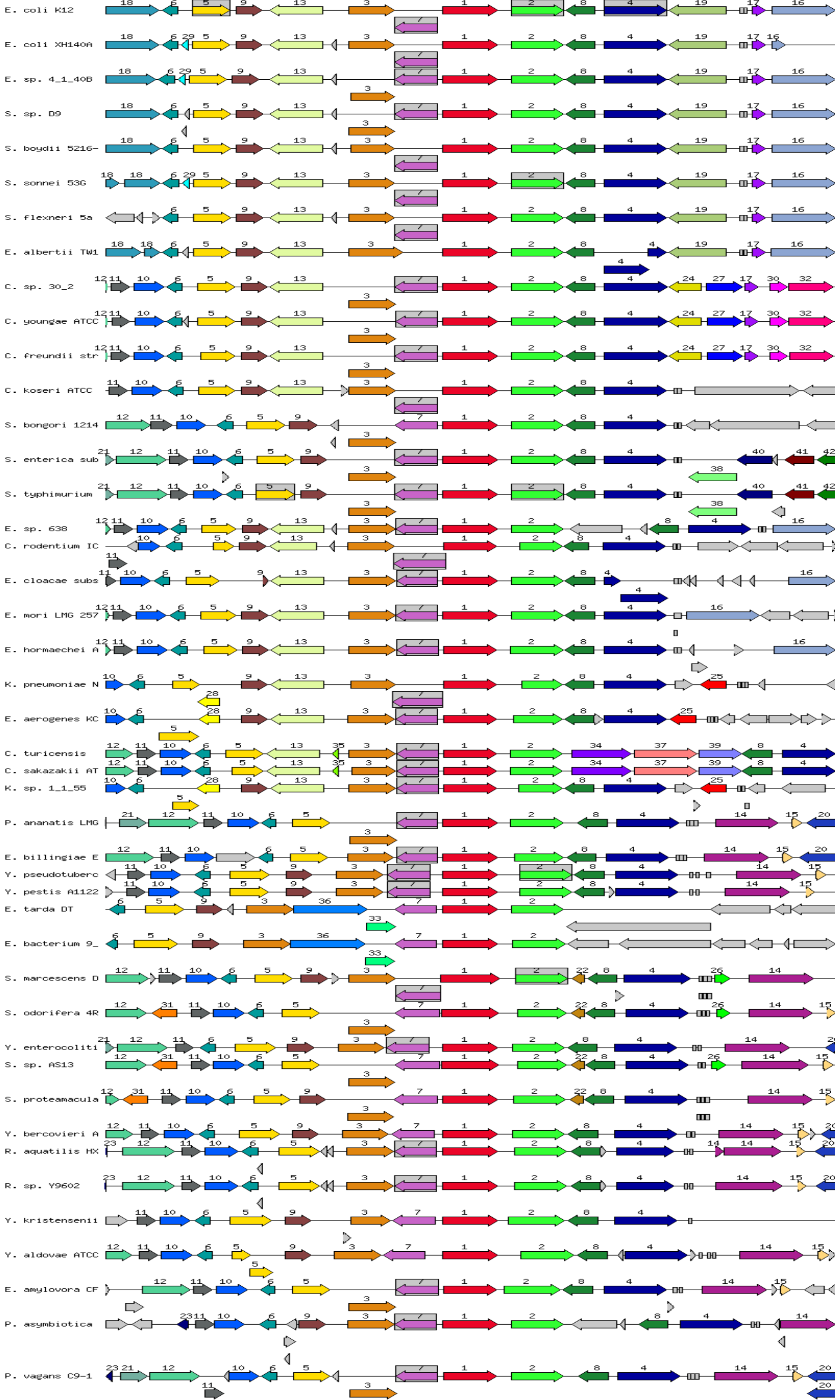

Fig. S1 The identifications for the genes encoding (marked by arrows): 1-YdhC, 7-YdhB, 3-PurR

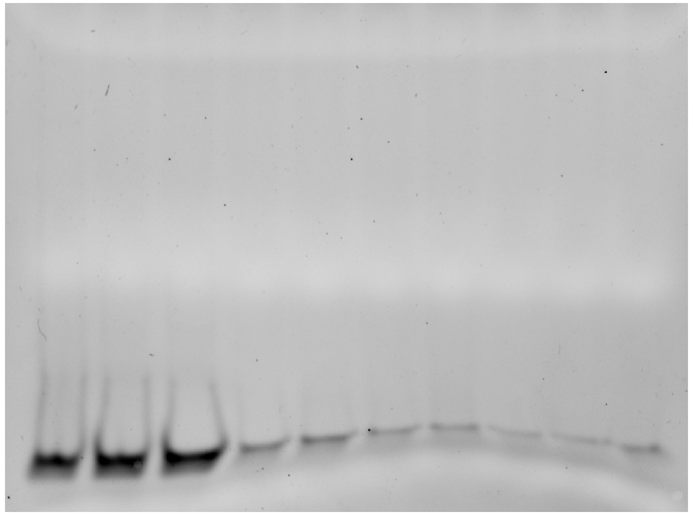

1, 2, 3, 4, 5, 6, 7, 8, 9, 10

Fig S3. The EMSA assay for the fluorescently labeled DNA pre-incubated 1 hour with PunR and separated on 6% polyacrylamide gel. The lines -1, 2, 3 represent 1.5, 0.8, 0 microM of PunR pre-incubated 1 hour with the negative control - GguR labeled DNA GTTGT(A/C)N(G/T)ACAAC from (Ref) in the presence of 1mM adenine . The lines 4, 5, 6, 7 represent pre-incubated PunR-predicted labeled DNA with PunR-0, 0.4, 0.8 and 1.5 microM in the presence of 1 mM adenine and lines 8, 9, 10 are PunR pre-incubated with the predicted DNA-binding site at the 1, 0.5, 0 microM in the presence of 10 mM adenine. The Tris-Glycine running buffer contains 0.25 mM adenine.

ydhC knockout strain

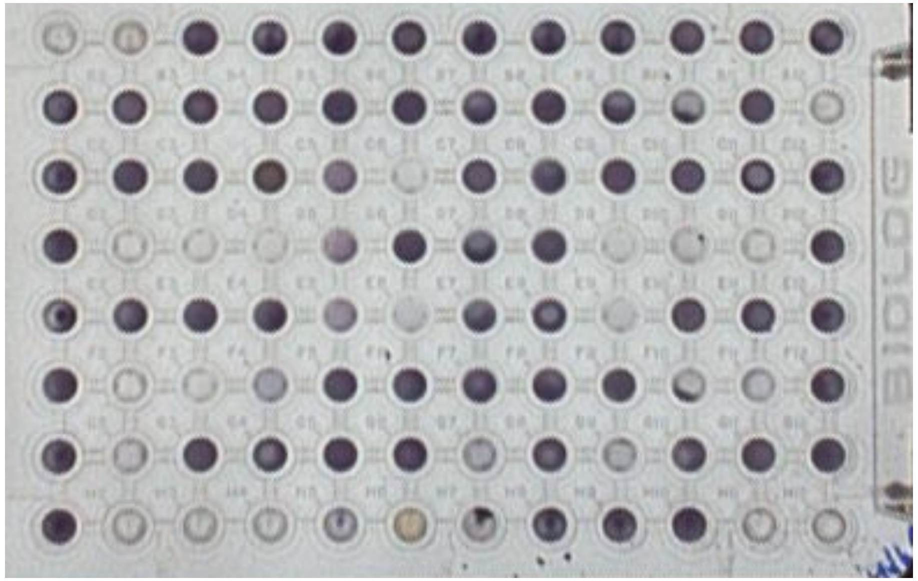

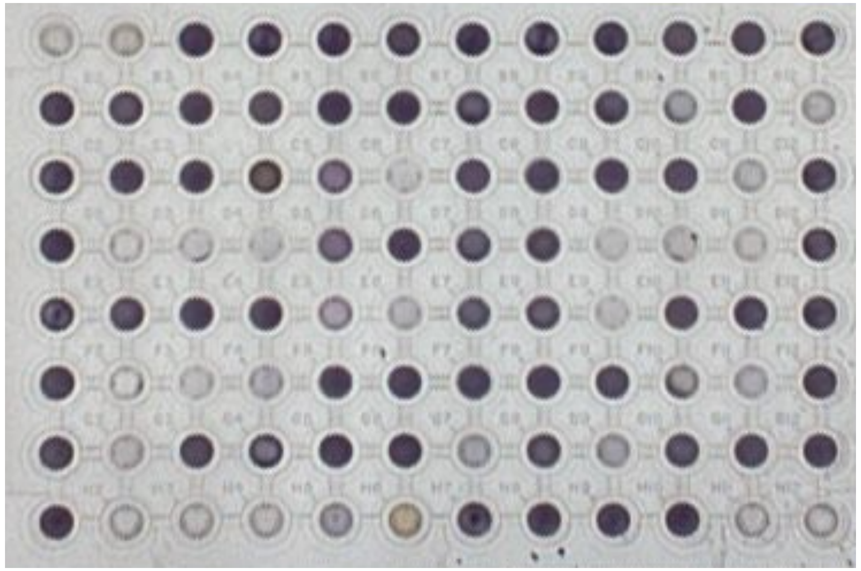

Fig. S4 The phenotype of the *E. co*li BW25113 NC and *punC* knockout strains in PM1.

b_number	The nearest gene
b0591	fepD
b1200	dhaR
b1241	adhE
b1499	ydeO
b1521	uxaB
b1638	pdxH
b1658	purR
b1659	ydhB
b1844	exoX
b2448	yffQ
b2632	yfjP
b2956	yggM
b3092	uxaC
b3175	secG
b3321	rpsJ

Table S1. The ChIP-exo detected the PunR occupied an 200bp region in the promoter/intergenic. Region for the listed genes.

Fig. S2 Multiple alignments of intergenic regions of the *ydhC/ydhB* genes in various groups of Enterobacteria

Enterobacteriaceae - 1

83333.1.peg.1645	Escherichia coli K12
457401.3.peg.399	Escherichia sp. 4_1_40B
216599.12.peg.1710	Shigella sonnei 53G
1038844.18.peg.786	Escherichia coli O104:H4 str. TY-2482
556266.3.peg.160	Shigella sp. D9
622.8.peg.966	Shigella dysenteriae CDC 74-1112
300268.11.peg.1907	Shigella boydii Sb227
373384.11.peg.1943	Shigella flexneri 5 str. 8401
208962.36.peg.1645	Escherichia albertii strain EC06-170
290338.8.peg.1409	Citrobacter koseri ATCC BAA-895
1173691.3.peg.3874	Citrobacter sp. A1
1288347.3.peg.145	Citrobacter freundii GTC 09479
500640.5.peg.1436	Citrobacter youngae ATCC 29220
701347.4.peg.2203	Enterobacter cloacae SCF1
1265617.3.peg.1260	Salmonella enterica subsp. enterica serovar Agona str. 36.H.00
35703.16.peg.2786	Citrobacter amalonaticus strain YG6
12149.1.peg.1388	Salmonella bongori 12149
575.4.peg.1602	Raoultella planticola strain CHB
571.108.peg.202	Klebsiella oxytoca strain CAV1335
1286170.3.peg.1018	Raoultella ornithinolytica B6
1134687.3.peg.2478	Klebsiella michiganensis strain SA2
936565.3.peg.5502	Klebsiella sp. OBRC7

```
                                                           ydhC <-
1173691.3.peg.3874      GGCCCGCCAGCCAGACTAAAAACCCTTTCCCAGGTTGCATcatcatttctcttttttttagcagtaga------acaatGccgct
1288347.3.peg.145       GGCCCGCCAGCCAGACTAAAAACCCTTTCCCAGGTTGCATcatcatttctcttttttttagcagtaga------acaatgccgct
500640.5.peg.1436       GACCCGCCAACCAGACTAAAAACCCTTTCCCAGGTTGCATcatcatttctctttt---agctataga------acaatgccgct
290338.8.peg.1409       GACCCGCCAGCCAGACTAAAAAACCTTTTCCGGGTTGCATaattgtttctcttttcc-agaagtaag------acattgccgct
83333.1.peg.1645        AACCCGCCAGCCAGACTAAAAATCTTTTCCCAGGTTGCATgtatatt-ctctaaatctctcaattaa------acaccgccgca
457401.3.peg.399        AACCCGCCAGCCAGACTAAAAATCTTTTCCCAGGTTGCATgtatatt-ctctaaatctctcaattaa------acaccgccgca
1038844.18.peg.786      AACCCGCCAGCCAGACTAAAAATCTTTTCCCAGGTTGCATgtatatt-ctctaaatctctcaattaa------acaccgccgca
556266.3.peg.160        AACCCGCCAGCCAGACTAAAAATCTTTTCCCAGGTTGCATgtatatt-ctctaaatctctcaattaa------acaccgccgca
216599.12.peg.1710      AACCCGCCAGCCAGACTAAAAATCTTTTCCCAGGTTGCATgtatatt-ctctaaatctctcaattaa------acaccgccgca
373384.11.peg.1943      AACCCGCCAGCCAGACTAAAAATCTTTTCCCAGGTTGCATgtatatt-ctctaaatctctcaattaa------acaccgccgca
622.8.peg.966           AACCCGCCAGCCAGACTAAAAATCCTTTCCCAGGTTGCATgtatatt-ctctaaatctctcaattaa------acaccgccgca
300268.11.peg.1907      AACCCGCCAGCCAGACTAAAAATCTTTTCCCAGGTTGCATgtatatt-ctctaaatctctcaattaa------acaccgccgca
208962.36.peg.1645      AACCCGCCAGCCAGACTAAAAATCTTTTCCCAGGTTGCATgtatatt-ctctaaattcctcaattaa------acaccgccgca
1265617.3.peg.1260      GACCGGCTAACCAGACTAAAAAACCTTTTCCAGGTTGCATatttgtt-tctctttcttatcagacag------acattgtcgca
12149.1.peg.1388        GACCGGCCAGCCAGACTAAAAAACCTTTTCCAGGTTGCATatttgtt-tctctttattatcaaacag------acactgccgca
35703.16.peg.2786       GCCCTGCCAGCCAGACTAAAAACCCTTTTCCTGGTTGCATagttgttctctaattttgattaatc--------acactggcgca
575.4.peg.1602          GTCCCGCCAGCCACACTAAAAATCCTTTCCCGGGTTGTTGCATaacattctctt---atagatgcag----gtatgatgccgca
1286170.3.peg.1018      GTCCCGCCAGCCACACTAAAAATCCTTTCCCGGGTTGTTGCATaacattctctt---ataggtgcag----gtatgatgccgca
1134687.3.peg.2478      GGCCAGCCAGCCAGACTAAAAATCCTTTCCCAGGTTGTTGCATaacattctctttttattgt-gcag----gtacgatggcgca
936565.3.peg.5502       GTCCAGCCAGCCAGACTAAAAATCCTTTCCCAGGTTGTTGCATcatcttctctttttattgttgcag----gtacgatggcgca
571.108.peg.202         GTCCGGCCAGCCAGACTAAAAATCCTTTCCCAGGTTGTTGCATatcattctctttttagtgatgcag----gtacgatggcgca
701347.4.peg.2203       AGCCTGCCAGCCAGCATAAAAATCCTTTACCAGGTTGCATcgtcatttctcattctcgttgcaggtgttcagttcagtgccgca
                          ** ** * ***   ****** * *** ** *****                                          * ***
```

```
                           -10                   -35                        YdhB
binding site             -> ydhB
1173691.3.peg.3874
gagtgtagtgagtgtaattctcgctgtgaaacgctaatatttgcttactgatttcaaaattttgcaggcagagaatATGTGGTC
1288347.3.peg.145
gagtgtagtgagtgtaattctcgctgtgaaacgctaatatttgcttactgatttcaaaattttgcaggcagagaatATGTGGTC
500640.5.peg.1436
gagtgtagtgagtgtaattcacgctgtgaaacgctaatatttgcttactgatttcaaaattttgcaggcagagaatATGTGGTC
290338.8.peg.1409
gagtgtagggagtgaaattctgcttgtgaaacgctaatatttggctactggattcaaaattttgcaggcagagaatATGTGGTC
83333.1.peg.1645
aagtgtagggagtgaaattagtgttgtgaaacggtaatatttgatgactggtttcaaaaattttgcaggcagagactATGTGGTC
457401.3.peg.399
aagtgtagggagtgaaattagtgttgtgaaacggtaatatttgatgactggtttcaaaaattttgcaggcagagactATGTGGTC
1038844.18.peg.786
aagtgtagggagtgaaattagtgttgtgaaacggtaatatttgatgactggtttcaaaaattttgcaggcagagactATGTGGTC
556266.3.peg.160
aagtgtagggagtgaaattagtgttgtgaaacggtaatatttgatgactggtttcaaaaattttgcaggcagagactATGTGGTC
216599.12.peg.1710
aagtgtagggagtgaaattagtgttgtgaaacggtaatatttgatgattggtttcaaaaattttgcaggcagagactATGTGGTC
373384.11.peg.1943
aagtgtagggagtgaaattagtgttgtgaaacggtaatatttgatgactggtttcaaaaattttgcaggcagagactATGTGGTC
622.8.peg.966
aagtgtagggagtgaaattagtgttgtgaaacggtaatatttgatgactgatttcaaaaattttgcaggcagagactATGTGGTC
300268.11.peg.1907
aagtgtagggagtgaaattagtgttgtgaaacggtaatatttgatgactgatttcaaaaattttgcaggcagagactATGTGGTC
208962.36.peg.1645
aagtgtagggagtgaaattcctgttgtgaaacggtaatatttgatgactggtttcaaaaattttgcaggcaaaaactATGTGGTC
1265617.3.peg.1260
gagtgtagggagtgaaattacggttgtgaaacgttaatatttgtccagtgaattcaaaattttgtaggcaaaaattATGTGGTC
12149.1.peg.1388
gagtgtagggagtgaaattaacgttgtgaaacgttaatatttgtccattgggttcaaaattttgcaggcatatataATGTGGTC
35703.16.peg.2786
gagtgtagggagtgaatttgggcttgtgaaacggtaatatttgtttattgcattcaaaaattttgtaggcagagattATGTGGTC
575.4.peg.1602
gagtgtagggagtgcattctggcttgtgaaacgctaatatttgctacatgcgttcaaaattttgcaggcagagaatATGTGGTC
1286170.3.peg.1018
gagtgtagggagtgcattctggcttgtgaaacgctaatatttgctacatgcattcaaaattttgcaggcagagaatATGTGGTC
1134687.3.peg.2478
gagtgtagggagtgcattaaggcttgtgaaacgctaatatttgtcagatgagttcaaaaattttgcaggcagagaaaATGTGGTC
936565.3.peg.5502
gagtgtagggagtgcattaaggcttgtgaaacgctaatatttgtcaggtgaattcaaaaattttgcaggcagagaaaATGTGGTC
571.108.peg.202
gagtgtagggagtgcattcaggcttgtgaaacgctaatatttgtcaggtgaattcaaaaattttgcaggcagagaaaATGTGGTC
701347.4.peg.2203
gagtgtagggagtgcatcctggcttgtgaaacgttaatatttgtcgaatgggttcaaaaattttgcaggcagagattATGTGGTC
                         ******* ***** *        ********* *********     **   *******
***** ***** *  ***********
```

1028307.3.peg.3552	Enterobacter aerogenes KCTC 2190
548.115.peg.1014	Enterobacter aerogenes strain CAV1320
883121.3.peg.2572	Klebsiella oxytoca 10-5246
1286170.3.peg.1018	Raoultella ornithinolytica B6
575.4.peg.1602	Raoultella planticola strain CHB
640131.3.peg.2253	Klebsiella variicola At-22
1134687.3.peg.2478	Klebsiella michiganensis strain SA2
701347.4.peg.2203	Enterobacter cloacae SCF1
1697388.3.peg.3561	Enterobacter tabaci strain CCUG 72520
290338.8.peg.1409	Citrobacter koseri ATCC BAA-895
546.84.peg.3727	Citrobacter freundii strain FDAARGOS_61
500640.5.peg.1436	Citrobacter youngae ATCC 29220
67829.4.peg.3727	Citrobacter murliniae strain P080C CL
295319.15.peg.1525	Salmonella enterica subsp. enterica serovar Paratyphi A str. ATCC 9150
12149.1.peg.1388	Salmonella bongori 12149
1095768.3.peg.1306	Enterobacter sp. JC163

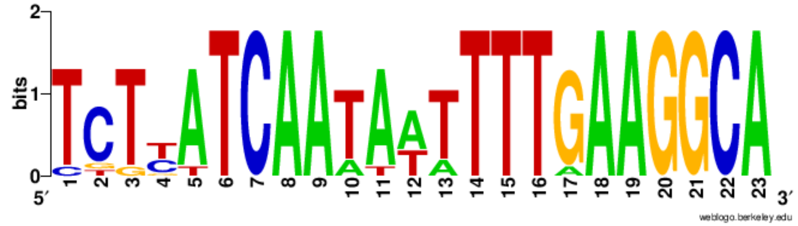

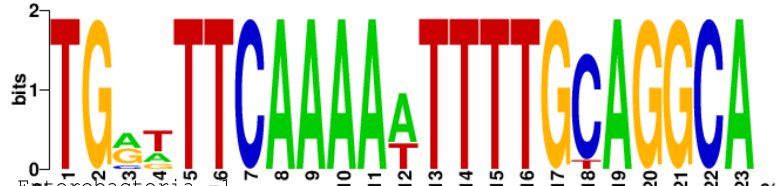

Enterobacteria -1

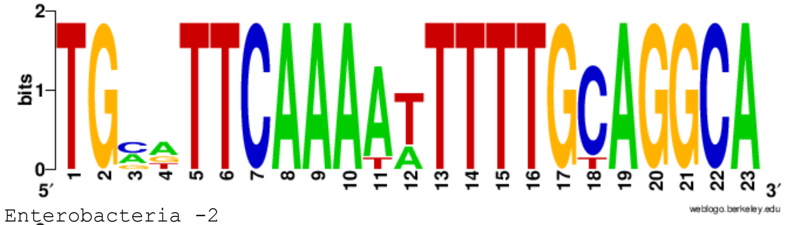

Enterobacteria -2

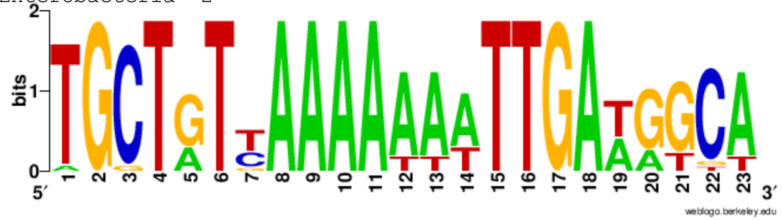

Enterobacteria -3

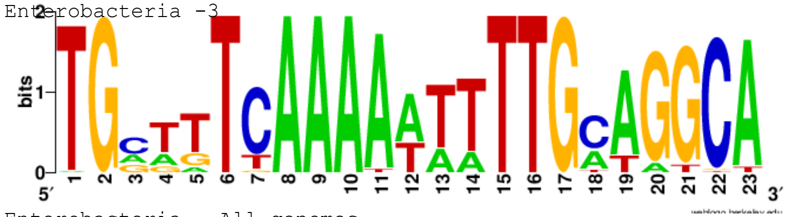

Enterobacteria - All genomes

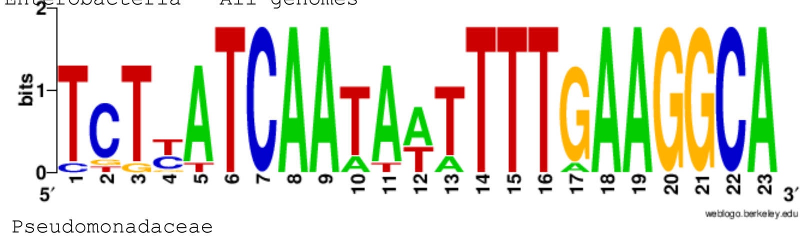

Pseudomonadaceae

```
                                   ydhC <-                                                              -10
701347.4.peg.2203       ATCCTTTACCAGGTTGCATcgtcatttctcatt-ctcgttgcaggtgttcagttcagtgccgcagagtgtag
1697388.3.peg.3561      ATCCTTTCCTGGGTTGCATtgttttattctcct-gaggaggcaggtgtacgt-----tggcgcagagtgtag
1028307.3.peg.3552      ATCCTTTCCCAGGTTGTTGCATaacgttctctttctactgatgcaggtatga-----tgccgcagagtgtag
548.115.peg.1014        ATCCTTTCCCAGGTTGTTGCATaacgttctctttctactgatgcaggtacga-----tgccgcagagtgtag
883121.3.peg.2572       ATCCTTTCCCGGGTTGTTGCATaacattctctt---ataggtgcaggtatga-----tgccgcagagtgtag
1286170.3.peg.1018      ATCCTTTCCCGGGTTGTTGCATaacattctctt---ataggtgcaggtatga-----tgccgcagagtgtag
575.4.peg.1602          ATCCTTTCCCGGGTTGTTGCATaacattctctt---atagatgcaggtatga-----tgccgcagagtgtag
640131.3.peg.2253       ATCCTTTCCCAGGTTGTGACATaacgttctcattttggagtacaggtacga-----tgccgcagagtgtag
1134687.3.peg.2478      ATCCTTTCCCAGGTTGTTGCATaacattctc-tttttattgtgcaggtacga-----tggcgcagagtgtag
546.84.peg.3727         ACCCTTTCCCAGGTTGCATCATcatttctcttttt--ttagcaatagaacaa-----tgccgctgagtgtag
500640.5.peg.1436       ACCCTTTCCCAGGTTGCATCATcatttctctt-----ttagctatagaacaa-----tgccgctgagtgtag
67829.4.peg.3727        ACCCTTTTCCAGGTTGCATCATcatttctctttt---taagcagtaaaacag-----tgtcgcagagtgtag
290338.8.peg.1409       AACCTTTTCCGGGTTGCATaattgtttctctttt---ccagaagtaagacat-----tgccgctgagtgtag
295319.15.peg.1525      AACCTTTTCCAGGTTGCATatttgtttctctttc---ttatcagacagacat-----tgtcgcagagtgtag
12149.1.peg.1388        AACCTTTTCCAGGTTGCATatttgtttctcttta---ttatcaaacagacac-----tgccgcagagtgtag
1095768.3.peg.1306      ACCCTTTTCCAGGTTGCATggt-aaatctctttt---taatgctgataacag-----ggctccagagtctaa
                        * ***** *  *****      *    *                                *   * **** **
```

```
                                                -35                      YdhB binding site  -> ydhB
701347.4.peg.2203       ggagtgcatcctggcttgtgaaacgttaatatttgtcgaatgggttcaaaaattttgcaggcagattATGTGGTCAGA
1697388.3.peg.3561      ggagtgcaaagcggcttgtgaaacgctaatatttgctgagtgcattcaaatttttgcaggcagaacATGTGGTCAGA
1028307.3.peg.3552      ggagtgtaatccgccttgtgaaacgctattatttgtcacatgcattcaaaaattttgcaggcagaatATGTGGTCTGA
548.115.peg.1014        ggagtgtaatccgccttgtgaaacgctattatttgtcacatgcattcaaaaattttgcaggcagaatATGTGGTCTGA
883121.3.peg.2572       ggagtgcattctggcttgtgaaacgctaatatttgctacatgcattcaaaattttgcaggcagaatATGTGGTCCGA
1286170.3.peg.1018      ggagtgcattctggcttgtgaaacgctaatatttgctacatgcattcaaaattttgcaggcagaatATGTGGTCCGA
575.4.peg.1602          ggagtgcattctggcttgtgaaacgctaatatttgctacatgcgttcaaaattttgcaggcagaatATGTGGTCCGA
640131.3.peg.2253       ggagtgcattgtggcttgtgaaacgctaatatttgtcacatgcattcaaaaattttgcaggcagaaaATGTGGTCTGA
1134687.3.peg.2478      ggagtgcattaaggcttgtgaaacgctaatatttgtcagatgagttcaaaaattttgcaggcagaaaATGTGGTCTGA
546.84.peg.3727         tgagtgtaattctcgctgtgaaacgctaatatttgcttactgatttcaaatttttgcaggcagaatATGTGGTCGGA
500640.5.peg.1436       tgagtgtaattcacgctgtgaaacgctaatatttgcttactgatttcaaaattttgcaggcagaatATGTGGTCGGA
67829.4.peg.3727        tgagtgaaattctcgctgtgaaacgctaatatttgtttactgatttcaaaatttttgtaggcagactATGTGGTCGGA
290338.8.peg.1409       ggagtgaaattctgcttgtgaaacgctaatatttggctactggattcaaaattttgcaggcagaatATGTGGTCAGA
295319.15.peg.1525      ggagtgaaattacggttgtgaaacgttaatatttgtccagtgaattcaaaattttgtaggcaaattATGTGGTCGGA
12149.1.peg.1388        ggagtgaaattaacgttgtgaaacgttaatatttgtccattgggttcaaaattttgcaggcatataATGTGGTCCGA
1095768.3.peg.1306      ggagtgcatccttgattgtgaaacgctaatatttggcggttgatttcaaaattttgcaggcagattATGTGGTCAGA
                         ***** *          ********* ** ******     **  ******   ***** ***** *  ******** **
```

Enterobacteriaceae - 3

630.33.peg.1425	Yersinia enterocolitica strain ATCC 9610
393305.7.peg.2324	Yersinia enterocolitica subsp. enterocolitica 8081
349966.5.peg.542	Yersinia frederiksenii ATCC 33641
28152.3.peg.1198	Yersinia kristensenii strain ATCC 33639
349965.6.peg.1196	Yersinia intermedia ATCC 29909
349967.4.peg.1011	Yersinia mollaretii ATCC 43969
527004.3.peg.123	Yersinia rohdei ATCC 43380
349968.5.peg.69	Yersinia bercovieri ATCC 43970
502800.6.peg.2008	Yersinia pseudotuberculosis YPIII
1084234.3.peg.4018	Yersinia pestis biovar Orientalis str. AS200901509
29486.45.peg.499	Yersinia ruckeri strain Big Creek 74
291112.3.peg.2119	Photorhabdus asymbiotica
615.109.peg.2214	Serratia marcescens strain UCI88
768490.3.peg.2144	Serratia sp. AS12
614.9.peg.259	Serratia liquefaciens strain FDAARGOS_125
667129.3.peg.3101	Serratia odorifera DSM 4582
399741.7.peg.2234	Serratia proteamaculans 568
82996.16.peg.4145	Serratia plymuthica strain A153
47917.12.peg.3337	Serratia fonticola strain GS2

Pseudomonadaceae

746360.3.peg.1750	Pseudomonas fluorescens WH6
1038924.3.peg.1593	Pseudomonas fluorescens SS101
321846.3.peg.1569	Pseudomonas simiae strain WCS417
1144885.3.peg.4026	Pseudomonas sp. R81
1037911.3.peg.1617	Pseudomonas fluorescens A506
96901.3.peg.1676	Pseudomonas synxantha BG33R
216595.4.peg.1849	Pseudomonas fluorescens SBW25
1125977.3.peg.5361	Pseudomonas sp. PAMC 25886
294.123.peg.1588	Pseudomonas fluorescens strain UK4
882211.3.peg.4099	Pseudomonas deceptionensis strain DSM 26521
