# Supplementary material for: Identification of a transcription factor, PunR, that regulates the purine and purine nucleoside transporter punC in E. coli
Source: Commun Biol. 2021 Aug 19;4:991. doi: 10.1038/s42003-021-02516-0 (PMC8376909; doi:10.1038/s42003-021-02516-0)
Supplement: Supplementary file 2 — Description of Additional Supplementary Files. [file 42003_2021_2516_MOESM2_ESM.pdf]

Description of Additional Supplementary Files

File name: Supplementary Data 1

Description: The punC, add and WT E. coli BW25113 growth raw data file.

File name: Supplementary Data 2

Description: RNA-Seq measured differentially expressed genes in the presence and absence of 2.5 mM adenosine compared for E. coli MG1655 wild type strain.
