# Supplementary material for: Identification of a transcription factor, PunR, that regulates the purine and purine nucleoside transporter punC in E. coli
Source: Commun Biol. 2021 Aug 19;4:991. doi: 10.1038/s42003-021-02516-0 (PMC8376909; doi:10.1038/s42003-021-02516-0)
Supplement: Supplementary file 5 — Reporting summary. [file 42003_2021_2516_MOESM5_ESM.pdf]

Corresponding author(s): COMMSBIO-21-0093

Last updated by author(s): Feb 9, 2021

Reporting Summary

Nature Research wishes to improve the reproducibility of the work that we publish. This form provides structure for consistency and transparency in reporting. For further information on Nature Research policies, see our Editorial Policies and the Editorial Policy Checklist.

Statistics

For all statistical analyses, confirm that the following items are present in the figure legend, table legend, main text, or Methods section.

n/a	Confirmed	
☐	☒	The exact sample size (*n*) for each experimental group/condition, given as a discrete number and unit of measurement
☐	☒	A statement on whether measurements were taken from distinct samples or whether the same sample was measured repeatedly
☐	☒	The statistical test(s) used AND whether they are one- or two-sided<br>*Only common tests should be described solely by name; describe more complex techniques in the Methods section.*
☐	☒	A description of all covariates tested
☐	☒	A description of any assumptions or corrections, such as tests of normality and adjustment for multiple comparisons
☐	☒	A full description of the statistical parameters including central tendency (e.g. means) or other basic estimates (e.g. regression coefficient) AND variation (e.g. standard deviation) or associated estimates of uncertainty (e.g. confidence intervals)
☐	☒	For null hypothesis testing, the test statistic (e.g. *F*, *t*, *r*) with confidence intervals, effect sizes, degrees of freedom and *P* value noted<br>*Give P values as exact values whenever suitable.*
☐	☒	For Bayesian analysis, information on the choice of priors and Markov chain Monte Carlo settings
☐	☒	For hierarchical and complex designs, identification of the appropriate level for tests and full reporting of outcomes
☐	☒	Estimates of effect sizes (e.g. Cohen's *d*, Pearson's *r*), indicating how they were calculated

Our web collection on statistics for biologists contains articles on many of the points above.

Software and code

Policy information about availability of computer code

Data collection	The RNAseq samples were collected in duplicates and differentially expressed genes were detected by the analysis discribed in Materials and Methods part.
Data analysis	The ICA (independent component analysis has been discribed (published previously by our Palsson group, UCSD).

For manuscripts utilizing custom algorithms or software that are central to the research but not yet described in published literature, software must be made available to editors and reviewers. We strongly encourage code deposition in a community repository (e.g. GitHub). See the Nature Research guidelines for submitting code & software for further information.

Data

Policy information about availability of data

All manuscripts must include a data availability statement. This statement should provide the following information, where applicable:

- Accession codes, unique identifiers, or web links for publicly available datasets
- A list of figures that have associated raw data
- A description of any restrictions on data availability

The RNAseq data generated during the study is presented GSM5267195 MG1655 ade, rep 1, GSM5267196 MG1655 ade, rep 2,GSM5267197 del_ydhB ade, rep 1

Field-specific reporting

Life sciences study design

All studies must disclose on these points even when the disclosure is negative.

Sample size	All samples were collected in duplicates and the growth curves (phenotype) measured in triplicates.
Data exclusions	No data were excluded from the analysis
Replication	The growth experiments were repeated 3 times.
Randomization	The allocation was random
Blinding	The investigators were blinded to group allocation.

Reporting for specific materials, systems and methods

We require information from authors about some types of materials, experimental systems and methods used in many studies. Here, indicate whether each material, system or method listed is relevant to your study. If you are not sure if a list item applies to your research, read the appropriate section before selecting a response.

Materials & experimental systems

n/a	Involved in the study
☒	☐ Antibodies
☒	☐ Eukaryotic cell lines
☒	☐ Palaeontology and archaeology
☒	☐ Animals and other organisms
☒	☐ Human research participants
☒	☐ Clinical data
☒	☐ Dual use research of concern

Methods

n/a	Involved in the study
☐	☒ ChIP-seq
☒	☐ Flow cytometry
☒	☐ MRI-based neuroimaging

ChIP-seq

Data deposition

☒ Confirm that both raw and final processed data have been deposited in a public database such as GEO.

☒ Confirm that you have deposited or provided access to graph files (e.g. BED files) for the called peaks.

Data access links<br>*May remain private before publication.*	GSE166465
Files in database submission	GSM5071452 ydhB_1 , GSM5071453 ydhB_2 Feb 09, 2023 approved GFF
Genome browser session<br>(e.g. UCSC)	https://github.com/SBRG/precise-db

Methodology

Replicates	the measurements in replicates have been provided
Sequencing depth	The 300bp reads for sequencing were provided
Antibodies	antibodies that specifically recognize the myc tag (9E10, Santa Cruz Biotechnology), and Dynabeads Pan Mouse IgG magnetic beads (Invitrogen)
Peak calling parameters	Peak calling was performed as previously described: Reading mapping-echo '#command line: ' $WHERE_BOWTIE $BOWTIE_OPTION $BOWTIE_REFERENCE $FILE_PE_LEFT '--un' $BOWTIE_UNALIGNED '>' $BOWTIE_OUTPUT<br>Peak calling:echo '#command line: ' 'python' $WHERE_MACE_DIR'preprocessor.py' $MACE_PREPOPT '-i' $FILE_BAM1,$FILE_BAM2 '-r' $FILE_SIZE '-o' $FILE_PREPROC<br>python $WHERE_MACE_DIR'preprocessor.py' $MACE_PREPOPT -i $FILE_BAM1,$FILE_BAM2 -r $FILE_SIZE -o $FILE_PREPROC<br>$WHERE_BOWTIE $BOWTIE_OPTION $BOWTIE_REFERENCE $FILE_PE_LEFT --un $BOWTIE_UNALIGNED > $BOWTIE_OUTPUT<br>from ChIP-exo were mapped onto the reference genome (NC_000913.2) using bowtie<br>with default options to generate SAM output files. MACE program was used to define peak candidates from biological duplicates for each experimental condition with sequence depth normalization
Data quality	To reduce false-positive peaks, peaks with signal-to-noise (S/N) ratio less than 1.5 were removed. The noise level was set to the top

	5% of signals at genomic positions because top 5% makes a background level in a plateau and top 5% intensities from each ChIP-exo replicates across conditions correlate well with the total number of reads
Software	Bowtie and MACE software were used.
